# Weighted Quantile Regression Forests for Bimodal Distribution Modeling: A Loss Given Default Case

**DOI:** 10.3390/e22050545

**Published:** 2020-05-13

**Authors:** Michał Gostkowski, Krzysztof Gajowniczek

**Affiliations:** 1 Department of Econometrics and Statistics, Institute of Economics and Finance, Warsaw University of Life Sciences-SGGW, 02-776 Warsaw, Poland; michal_gostkowski@sggw.edu.pl; 2 Department of Artificial Intelligence, Institute of Information Technology, Warsaw University of Life Sciences-SGGW, 02-776 Warsaw, Poland

**Keywords:** bimodal distribution, loss given default, machine learning, weighted quantile regression forests

## Abstract

Due to various regulations (e.g., the Basel III Accord), banks need to keep a specified amount of capital to reduce the impact of their insolvency. This equity can be calculated using, e.g., the Internal Rating Approach, enabling institutions to develop their own statistical models. In this regard, one of the most important parameters is the loss given default, whose correct estimation may lead to a healthier and riskless allocation of the capital. Unfortunately, since the loss given default distribution is a bimodal application of the modeling methods (e.g., ordinary least squares or regression trees), aiming at predicting the mean value is not enough. Bimodality means that a distribution has two modes and has a large proportion of observations with large distances from the middle of the distribution; therefore, to overcome this fact, more advanced methods are required. To this end, to model the entire loss given default distribution, in this article we present the weighted quantile Regression Forest algorithm, which is an ensemble technique. We evaluate our methodology over a dataset collected by one of the biggest Polish banks. Through our research, we show that weighted quantile Regression Forests outperform “single” state-of-the-art models in terms of their accuracy and the stability.

## 1. Introduction

Based on the Basel II and Basel III Accords [1,2] banks in the EU need to keep a specified amount of capital to reduce the impact of their insolvency. The banks may choose one of the two following options: the Standardized Approach or the Internal Rating Approach. The Internal Rating Approach enables institutions to develop their own models for the purpose of calculating the required equity for credit collateralization. As part of the Basel II and Basel III Accords, three parameters were determined. The first parameter is probability of default (*PD*), the next one is loss given default (*LGD*) and the last one is exposure at default (*EAD*). All three parameters play an important role in the measurement and management of credit risk.

In the beginning, researchers and practitioners were focused only on the first parameter, probability of default; however, in recent years, more attention has been paid to loss given default [3]. It can be stated that if the *LGD* is not determined correctly, the bank needs to hold more capital and, at the same time, less money may be devoted to investment. The correct estimation of *LGD* may lead to a healthier and riskless allocation of capital.

It can be observed that one of the most popular models for *LGD* modeling is Ordinary Least Squares (OLS) [4]. When considering a very specific distribution of *LGD* parameters, the beta distribution model also is considered [5]. Non-parametric methods (such as regression trees or neural networks) are also considered in the modeling of *LGD* values. However, it should be noted that most research papers consider only the prediction of the mean, without considering the entire *LGD* distribution. Since the *LGD* values are usually associated with full loss or full recovery, the *LGD* distribution has a bimodal shape and predicting the mean value may be misleading. To overcome this fact, quantile regression was adopted to model *LGD* distribution [3,6].

However, any “single” model is often less accurate than an ensemble of models, which may lead to inferior out-of-sample performance. Ensemble models constitute a relevant function in data analytics and can be created in a variety of ways. Alongside such techniques like bagging, boosting and simple voting, one can employ the Random Forest algorithm (RF). The purpose of RF is to combine forecasts of several base models (decision trees) in order to improve reliability and generalizability over a single model [7]. There is strong evidence that a single model can be outperformed by an ensemble of models combined to reduce bias, variance or both.

For regression problems, Random Forests give an accurate approximation of the conditional mean of a response variable. It can be shown that RF provides information about the full conditional distribution of the response variable (such as *LGD*), not only about the conditional mean. Conditional quantiles can be inferred with quantile Regression Forests (qRF), a generalization of standard RF [8]. Quantile Regression Forests give a nonparametric and accurate way of estimating conditional quantiles for high-dimensional predictor variables.

As already mentioned, ensemble algorithms aim to combine many diverse base predictive models; however, there is always a question what influence each model should have on the final prediction. The simplest approach is to give equal weight to each learner. However, numerous studies have shown that a weighted ensemble can provide better forecasting results [7]. For this reason, this research presents a novel weighted quantile Regression Forest algorithm (wqRF), which assigns higher weights in quantile estimation to those base learners with better generalization abilities. To prove the effectiveness of our methodology, we compare the proposed approach with other state-of-the-art benchmarking methods, including ordinary least squares, quantile regression, and standard quantile Regression Forests.

In light of the aforementioned reasons and motivations, using the dataset collected by one of the biggest Polish banks [9], we aim to answer the following research questions:

- To what extent is it possible to model and predict future values of the loss given default?
- Does the proposed weighting method introduce improvements to the standard quantile Regression Forest algorithm?
- Which modeling method best determines the loss given default in terms of the performance on a new unseen dataset?

The remainder of this paper is organized as follows: Section 2 provides an overview of similar research problems in relation to loss given default modeling, machine learning, the Random Forest algorithm and weighting methods. In Section 3 and Section 4, the theoretical framework of the bimodal distribution and the weighted quantile Regression Forest algorithm is presented. Section 5 outlines the experiments and presents the discussion of the results. The paper ends with concluding remarks in Section 6.

## 2. Preliminaries

### 2.1. Loss Given Default Modeling

In the literature, several definitions of the loss given default were proposed. *LGD* can be defined as the percentage of total exposure at the time of the default that cannot be recovered [10]. In other words, *LGD* is a percentage of nonrecoverable debt in relation to *EAD* [11]. Sometimes, *LGD* can be expressed in relation to recovery rate (*RR*) [12]:

(1) LGD=1−RR

It should be noted that *LGD* distribution is not normal [13]. Typically, *LGD* values are bounded by zero and one, but in certain situations values outside the specified range can be observed. Moreover, *LGD* values are very often close to one (full loss) or very close to zero (full recovery). Such a distribution may be expressed as a ‘two-humped’ distribution [14]. The bimodality of *LGD* distribution makes parametric modeling difficult to use [15] and using an “average” in terms of *LGD* may be misleading.

The main goal of *LGD* modeling is to generate simulated *LGD*s that are very close and highly correlated with historical *LGD*s The problems with modeling *LGD*s are related to the data used in the exercise, the proper choice of the model parameters and the limitations of the models [16]. In recent years, different approaches were proposed to model *LGD* values. Dermine and de Carvalho [17] incorporated one of the most popular statistical models, i.e., the log-log model. They used micro-data provided by one of the largest private banks in Portugal. The dataset includes 10,000 short-term loans granted by given banks to the small and medium sized enterprises (SME) sector. The data were collected over the period 1995–2000. Using this model, they identified several explanatory variables that may have an impact on the final values of the recovery rate (in this paper, instead of using loss given default, they analyzed recovery rates).

Similar research was conducted by Hamerle et al. [12]. They used a dataset from Moody’s Default Risk Service. In this dataset, information about 2000 defaulted debt obligations were collected from 1983 to 2003. Significant portions of information about debt obligations are from the USA. They incorporated a simple linear regression, but *LGD* was transformed using logistic transformation.

Chalupka and Kopecsni [15] considered another approach. The *LGD* values were mapped to a number of *LGD* grades. Using this approach, the distribution of the *LGD* variable in each grade is close to the normal distribution. They used grades proposed by Moody: *LGD*1 is 0–10%, *LGD*2 is 10–30%, *LGD*3 is 30–50%, *LGD*4 is 50–70%, *LGD*5 is 70–90% and *LGD*6 is 90–100% [15]. They used micro-data containing a set of firm loans from an anonymous Czech commercial bank. In the paper, several methods were analyzed and compared, such as generalized linear models for ordinal responses and fractional responses using beta inflated distribution. The research showed that models assuming beta distribution achieved slightly worse results in comparison to the other methods analyzed in the paper.

Similar research assuming the beta distribution of *LGD* was conducted by Huang and Oosterlee [18]. They compared various kinds of beta regression for *LGD* modeling and confirmed that beta regression can be successfully used in *LGD* modeling. Similar results were presented by Bellotti and Crook [19]. All mentioned models can be classified as parametric models and functional forms need be specified before modeling. To overcome this fact, non-parametric methods were also used in *LGD* modeling.

Hurlin et al. [20] compared six competing *LGD* models, such as (1) logistic regression, (2) regression trees, (3) Random Forests, (4) gradient boosting, (5) artificial networks and (6) support vector machines. They used a sample of almost 10,000 defaulted credit and leasing contracts collected by the bank of a world-leading automotive company. They covered Brazilian customers defaulted between 2011 and 2014. They proposed a new loss function and compared this function with a traditional approach focused only on forecast errors. A similar set of models was the subject of an analysis presented by Qi and Zhao [4] and Bastos [21]. They proved that non-parametric models perform better in comparison to parametric models. Hartmann-Wendels et al. [22] provided an extensive comparison of regression and tree models. They used a dataset provided by German leasing companies.

Yao et al. [23], with success, used support vector regression to model *LGD* values. They used Moody’s Ultimate Recovery Database with more than 6000 default debt instruments. The focus was on corporate bonds from 1985 to 2012. They considered several modifications of support vector regression and the results show that support vector regression outperforms other statistical models.

Krüger and Rösch [3] proposed a quantile regression to obtain the entire *LGD* distribution. In this paper, a dataset of American defaulted loans to small and medium enterprises from Global Credit Data were used. They used data from 2009 to 2013. Additionally, the use of *LGD* values instead of *RR* values was considered. The authors proved that quantile regression is very well suited to model distributions with bimodal shapes.

### 2.2. Machine Learning and Random Forests

Random Forests [24] are an ensemble supervised machine learning technique that emerged at the beginning of this millennium as an extension of random decision forests [25,26]. Machine learning techniques have applications in many areas of data analysis, such as describing data (distributions estimation) or analyzing data using the unsupervised approach. Widely solved problems in those domains are clustering, including algorithms like hierarchical clustering, k-means [27,28], dimensionality reduction techniques including non-negative matrix factorization or principal component analysis [29] and association and sequential rule-mining algorithms (CM-SPADE, Apriori, PrefixSpan, Eclat) [30].

Another approach, called supervised paradigm, aims to explore past data and generate conclusions or trends for future prediction [31]. The main objective in supervised learning is to estimate a function that maps an input (often called as independent variables or features) to an output (dependent variable or target) based on example input-output pairs. It infers a function from labeled training data sample consisting of a set of training examples that can be further used for mapping new examples (from validation or test samples). Most commonly used algorithms in this context are (problem-wise classification) supervised and semi-supervised learning, including naïve Bayes, decision trees, k-nearest neighbor, support vector machine, artificial neural network or boosting [32,33], structured prediction techniques, including random field, and Bayesian networks [34].

Generally, supervised learning analyzes two problems in terms of the nature of the output variable [35], i.e., regression aims at predicting a continuous quantity, while classification identifies a set of predefined categories/classes.

Many studies have shown that an ensemble is often more accurate than any single model in the ensemble [36,37] due to its ability to handle highly non-linear data, parameter tuning simplicity, robustness to noise and learning speed (ability to process the data in parallel). Bagging [38] and boosting [39,40] are two widely used ensemble algorithms incorporating re-sampling techniques to obtain different (diverse) training samples for each of the classifiers. The first technique generates classifiers that are independent of each other, while the latter produces dependent classifiers [41]. The theoretical and empirical research related to ensembles have shown that an ideal ensemble consists of highly correct classifiers that disagree as much as possible [41].

There have been some studies on weighted ensembles in the literature; however, none of them deal with quantile regression problems. Xuan et al. [42] proposed refined weighted Random Forests, where weights are calculated based on all training data including in-bag (INB) data and out-of-bag (OOB) data. Next, they use a margin between the probability of foreseeing a real class and a false class label. The margin measures the extent to which the expected number of votes for the relevant class exceeds the expected number of votes for another class. Kuncheva and Rodríguez [43] investigated the optimality conditions for four combination methods: majority vote, weighted majority vote, the recall combiner and naïve Bayes. Pham and Olafsson [44] weighted each tree by replacing the regular average with a Cesaro average. Weighting based on the variable performance and importance was developed by Byeon et al. [45] and Booth et al. [46]. Finally, Utki et al. [47] created a weighted random survival forest by assigning weights to survival decision trees or to their subsets.

## 3. Bimodal Distribution

**Figure 1:**
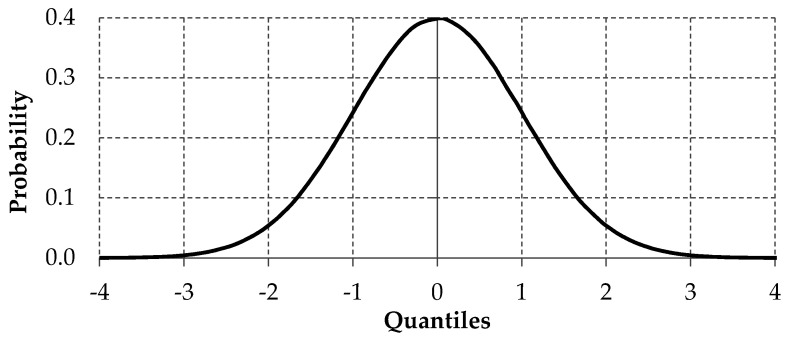
The normal distribution example.

Unimodal distribution is one of the most popular assumptions used in empirical modeling. Unimodal means that the given distribution has only one mode [48] and a typical example of unimodal distribution is normal distribution (see Figure 1). Moreover, normal distribution can be classified as symmetric distribution, but in many empirical analyses, symmetric assumption is too strong and other asymmetrical distributions are utilized (for example, Snedecor’s F distribution).

**Figure 2:**
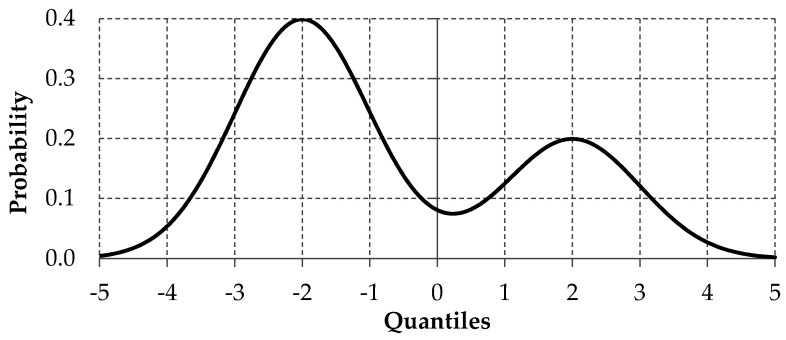
The example of bimodal distribution.

In empirical analyses, analyzed data are frequently bimodal and cannot be modeled by unimodal distributions [49]. Bimodality means that a given distribution has two modes and a large proportion of observations with large distances from the middle of the distribution [50]. It may provide important information about the nature of the analyzed variable (for example, the polarization of opinions if the variable represents a preference). Additionally, bimodality may indicate that the analyzed sample comes from two or more “overlapping” distributions or the analyzed sample is not homogenous (see Figure 2). In such a situation, a deep dive analysis should be performed to discover the reason for bimodality and, in the case of bimodal distributions, summary statistics such as median and mean can be deceptive and measures such as kurtosis and standard deviation will be extremely large in comparison to unimodal distribution. The extension of bimodal distribution creates multimodal distribution.

Different bimodal datasets were presented by many authors: Chatterjee et al. [51], Famoye et al. [52], Bansal [53]. Bimodal distribution comes from a mixture of two different unimodal distributions and one of the most popular bimodal distributions is two-component normal mixture distribution [54]. The probability density function of a mixture of two-component normal distributions is as follows [55]:

(2) $$f\left(x,p\right)=p\;f_{1}\left(x\right)+\left(1-p\right)\;f_{2}\left(x\right),$$

where, for i=1,2:

(3) $$f_{i}\left(x\right)=\frac{1}{\sigma_{i}\sqrt{2\pi }}e^{-\frac{1}{2}{\left(\frac{x-\mu_{i}}{\sigma_{i}}\right)}^{2}},$$

with 0<p<1. The density f(x,p) may have more than one mode. Additionally, a mixture of two-component normal distributions is closely related to the Exponential Power Family [54]. Mixture modeling has been a favorable model-based technique in supervised and unsupervised clustering problems [56].

Moreover, bimodality has an important impact on econometric analysis. One of the crucial decisions in the assessment of econometric models is statistical significance. This can be assessed using one of the most popular tests, Student’s *t*-test, which assumes that the distribution of the modeling variable is normal. It implies that the modeling variable has one mode [57]. In the case of modeling variables with two or more modes, Student’s *t*-test may be misleading.

## 4. Weighted Quantile Regression Forests

The Random Forest algorithm introduced by Breiman [24] works by constructing many decision trees and outputting the prediction of the individual trees by utilizing a training sample (training dataset D) of n observations, target variables Y∈R (where $y=\left(y_{1},\ldots y_{i},\ldots ,y_{n}\right)$ is an empirical realization of Y) and p predictor variables $X=\left\{X_{1},\ldots ,X_{j}\ldots ,\;X_{p}\right\}$, where each feature $X_{j}$ can take a value from its own set of possible values $\chi_{j}$ (moreover, $x_{i}=\left(x_{i1},\ldots ,x_{ip}\right)$ is an empirical realization of X). The main objective of each decision tree is to find a model (DT) for predicting the values of Y from new X values [7]. In theory, the solution is simply a recursive partition of X space into the disjointed rectangular subspace of χ (eventually achieving a final node called the leaf, denoted as $R_{l}\subseteq \chi ,\;l=1,\ldots ,L$) in such a way that the predicted ($\hat{Y}$) value of Y minimizes the total impurity of its child nodes (it is usually assumed that each parent node has two children, i.e., binary tree are considered). One of the first and widely used decision tree algorithms is the classification and regression tree (CART) [58], employing a measure of node impurity based on the distribution of the observed y values in the node by splitting a node that minimizes the total impurity of its two child nodes, defined by the total sum of squares [7]:

(4) $$TSS=\overset{n}{\underset{i=1}{\sum }}{\left(y_{i}-\bar{y}\right)}^{2},$$

where $\bar{y}$ denotes the average value of vector y over all observations belonging to a particular node. The process is applied recursively to the data in each child node. Splitting stops if the relative decrease in impurity is below a pre-specified threshold.

The prediction of a single tree DT for a new data point $x_{new}$ is obtained by averaging the observed values in l-leaf. Let the weight vector w be given by a positive constant if observation $x_{i}$ is part of l-leaf and zero if it is not. The weights add up to one, and thus [8]:

(5) $$w_{i}=\frac{I\left(x_{i}\in R_{l}\right)}{\#\left\{j:x_{j}\in R_{l}\right\}},$$

then, the prediction can be computed as the weighted average of the target values y:

(6) $${\hat{Y}}^{DT}=\overset{n}{\underset{i=1}{\sum }}w_{i}y_{i}.$$

There are various stopping criteria controlling the growth of the tree such as the minimum number of observations that must exist in a node in order for a split to be attempted, the minimum number of observations in any terminal node (leaf), or the maximum depth of any node of the final tree [7]. To achieve good generalization ability (i.e., small error rate on the unseen examples), the tree first grows in an overly large size and then it is pruned to a smaller size to minimize the misclassification error. CART employs a 10-fold (default) cross-validation [7].

The training algorithm for RF applies the general technique of bootstrap aggregating [38], also called bagging, to the base learners (decision tree). Given a training dataset D of size n, bagging generates m new training sets $D_{k}$, each of size $n^{\prime }$, by sampling from D uniformly and with a replacement [7]. By sampling with a replacement, some observations may be repeated in each $D_{k}$. If $n^{\prime }=n$, then, for large n, the dataset $D_{k}$ is expected to have the fraction $1-\frac{1}{e}\approx 63.2\%$ of the unique examples of D, with the rest being duplicates. The m (1,…k,…,m) models are fitted using the above m bootstrap samples and combined by averaging [7]:

(7) $${\hat{Y}}^{RF}=\overset{n}{\underset{i=1}{\sum }}m^{-1}\overset{m}{\underset{k=1}{\sum }}{\hat{Y}}_{ik}^{DT}.$$

This bootstrapping procedure leads to better model performance because it decreases the variance in the model without increasing the bias. This means that although the predictions of a single tree are highly sensitive to noise in its training dataset, the average of many trees is not as long, as the trees are not correlated.

The above procedure describes the original bagging algorithm for decision trees. The RF algorithm has an additional modification, i.e., it uses a modified algorithm, called a Random Decision Tree, that selects a random subset of the features mtry at each candidate split in the learning process. This process is sometimes called feature bagging [7]. The reason for doing this is the correlation of the trees in an ordinary bootstrap sample: if one or a few features are very strong predictors of the response variable (target output), these features will be selected in many of the m trees, causing them to become correlated. Typically, for a regression problem with p features, floor(p/3) features are used in each split [7].

Each tree within the forest is built to its maximum size, i.e., without pruning. The evaluation of the model performance on the training dataset is often replaced by an out-of-bag sample. This is a method of measuring the prediction error on the remaining 36.8% observations not observed in the bootstrap sample (INB). OOB is the mean prediction error using only the trees that did not have a particular observation in their bootstrap sample [7].

According to Equation (7), it can be shown that Regression Forests approximate the conditional mean E(Y|X=x). It can be suspected that RF delivers not only a good approximation of the conditional mean, but also that it does this to the full conditional distribution. The conditional distribution function of Y, given X=x, is given by:

(8) F(y|X=x)=P(Y≤y|X=x)=E(I(Y≤y)|X=x),

where I is the indicator function, which, for the Regression Forests, gives an approximation of any parameter (e.g., quantile) of the conditional distribution:

(9) $$\hat{F}\left(y|X=x\right)=\overset{n}{\underset{i=1}{\sum }}m^{-1}\overset{m}{\underset{k=1}{\sum }}I\left({\hat{Y}}_{ik}^{DT}\leq y\right).$$

Based on the above equation and for a given a probability α, we can estimate the quantile $Q_{\alpha }$ as:

(10) $${\hat{Q}}_{\alpha }\left(Y|X=x\right)=inf\left\{y:\hat{F}\left(y|X=x\right)>=\alpha \right\}.$$

The quantiles give more complete information about the distribution of Y as a function of the predictor features X than the conditional mean alone.

The original quantile Regression Forests implementation aggregates tree-level results equally across trees. In this article, we present the implementation of the standard qRF algorithm to build trees in the forest, but we use performance-based weights for quantile estimation, so that trees with better performance weigh more:

(11) $${\hat{Q}}_{\alpha }^{weighted}\left(Y|X=x\right)=\overset{n}{\underset{i=1}{\sum }}\frac{1}{\sum_{k=1}^{m}w_{k}}\overset{m}{\underset{k=1}{\sum }}w_{k}I\left({\hat{Y}}_{ik}^{DT}\leq y\right).$$

Since weights are based on the performance of a certain tree, applying weights to the same dataset from which they were calculated would bias the prediction error assessment [7]. Because of this, the predictive performance of each tree is assessed using observations from OOB samples as a weighted root mean square error (wRMSE):

(12) $$wRMSE=\sqrt{\underset{i\in OOB}{\sum }w_{i}^{obs}{\left(y_{i}-{\hat{Y}}_{i}^{DT}\right)}^{2}}.$$

It should be noted that some observations are more difficult to predict than others. Due to this, some learning algorithms (e.g., AdaBoost) incorporate observation weighting [59] and, for the same reason, Equation (12) contains weight $w_{i}^{obs}$ associated with each observation. Observation weighting can be also considered as a fairness problem [60], which is by definition the absence of any prejudice or favoritism towards an individual or a group based on their intrinsic or acquired traits in the context of decision making. In qRF, some trees may have a better performance on an OOB sample because they have been trained on the same observations, i.e., the INB sample. Such observations are difficult to correctly predict by trees that did not have them in the INB used for training [7]. Due to this, to assess the performance of each tree, we introduce weight based on the observations in the out-of-bag sample:

(13) $$w_{i}^{obs}=\frac{1}{\left|OOB_{i}\right|}\underset{k\in OOB_{i}}{\sum }\left|y_{i}-{\hat{Y}}_{ik}^{DT}\right|.$$

where $OOB_{i}$ stands for a set where i-th observation from k-th base tree belongs to the out-of-bag sample. For example, if $w_{i}^{obs}=0$ (meaning that each tree correctly predicts the true value), the observation is practically omitted.

Finally, to calculate the final weights used in Equation (11), we incorporate the ranked weights approach, comprised of two stages [7]:

- Ranking the pre-defined criterion (wRMSE);
- Weighting the criteria from their ranks using some rank order weighting approach.

For better understanding of this approach let us assume that we have a list of m prioritized (ranked) criteria, where each criterion k has a rank $r_{k}$ (k=1,…,m). The goal is to select and rank a set of m criteria that seems to be relevant, giving each k-th criterion a rank $r_{k}$. The rank is inversely related to weight, which means that the first rank $r_{1}=1$ denotes the highest weight (best tree), whilst rank $r_{m}=m$ denotes the lowest weight (worst tree). Many authors suggest various approaches for assigning weights based on a given criterion, e.g., rank reciprocal (inverse), rank sum (linear) and rank exponent weights. In this paper, we assume that weights should be squared [61]:

(14) $$w_{k}=\frac{{\left(m-r_{k}+1\right)}^{2}}{\sum_{k=1}^{m}{\left(m-r_{k}+1\right)}^{2}},$$

where $r_{k}$ is the rank of the k-th tree. The complete weighted quantile Regression Forest pseudocode is summarized in Algorithm 1 (below).
**Algorithm 1.** Weighted quantile Regression Forest algorithm pseudocode.**input**: Number of Trees (m), random subset of the features (mtry), training dataset (D), probability α for quantile estimation**output**: weighted quantile Regression Forests (wqRF)1:   wqRF is empty2:   **for each**
k = 1 to m
**do**3:     $D_{k}$ = Bootstrap Sample (D)4:     $DT_{k}$ = Random Decision Tree ($D_{k}$, mtry)5:     wqRF = $wqRF\cup DT_{k}\;$
6:   **end**7:   **for each**
i = 1 to n
**do**8:     Compute $w_{i}^{obs}$ using Formula (13)9:   **end**10:   **for each**
k = 1 to m
**do**11:     Compute $wRMSE_{k}$ using Formula (12)12:   **end**13:   **for each**
k = 1 to m
**do**14:     Compute $w_{k}$ using Formula (14)15:   **end**16:   Compute final prediction ${\hat{Q}}_{\alpha }^{weighted}\left(Y|X=x\right)$ using Formula (11)17:   **return**
wqRF


## 5. Empirical Analysis

### 5.1. Benchmarking Methods

In this paper, three benchmarking methods were selected and compared in terms of their accuracy and stability. The first selected method was ordinary least squares. The second method was a quantile regression model. Quantile regression models enable us to estimate the conditional quantiles of dependent variables and can be treated as an extension of the ordinary least squares method. Additionally, quantile regression is more robust against non-typical observations. The last selected method was a standard quantile Regression Forest.

### 5.2. Numerical Implementation

The numerical experiment was prepared using R programming language [62] on a Ubuntu 18.04 operating system on a personal computer equipped with Intel Core i7-9750H 2.6 GHz processor (12 threads) and 32 GB RAM. The *missRanger* package was used for the imputation of missing values, where each variable is imputed by predictions from a Random Forest, using all other variables as covariables. The feature selection algorithm is based on the *hclust* function, implementing a hierarchical clustering algorithm. Quantile regression models were estimated using *qr* function implemented in the *quantreg* library, which provides estimations and inference methods for models of conditional quantiles. Models estimated based on the ordinary least squares were trained using the *lm* function. The core for the wqRF is the *quantregForest* package, implementing the quantile Regression Forests for the estimation of conditional quantiles [8]. This package is particularly well suited for high-dimensional data. Predictor variables of mixed classes can be handled as well. Weighted quantiles were estimated using the *wtd.quantile* function provided by the *Hmisc* package.

### 5.3. Data Characteristics

In this paper, the data collected by one of the biggest Polish banks was used. The data were collected for overdraft products in the small- and medium-sized enterprises (SME) sector. Overdrafts are revolving loans that relate to current accounts with fixed monetary limits for individuals or enterprises [9]. The importance of overdraft products in the banking sector was explored by Williams [63]. Potential variables were identified from different sources such as customer relationship history, transaction history, customer segmentation or credit bureau ratings. The set of potential variables was prepared to cover all customer characteristics monitored in practice. Finally, eleven different data sources were identified.

**Figure 3:**
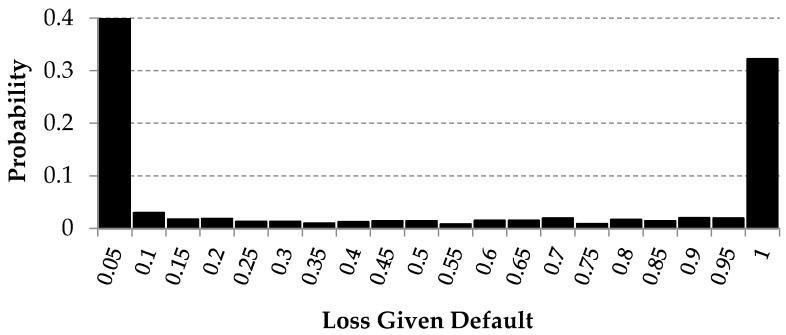
Histogram of the loss given default variable.

After determining a potential data source, the potential variables and the analytical base table were created. The data were collected from different sources and this resulted in a large number of gaps. The final dataset contained 292 variables and 1129 observations. All *LGD* values were in the default range, which means between zero and one. The distribution of the *LGD* variable is presented below (see Figure 3). Based on the summary statistics, it can be stated that more than 25% of observations are equal to zero. On the other hand, more than 25% of observations are equal to one. The mean value is very close to 0.5, but the median is slightly lower (see Table 1).

**Figure 4:**
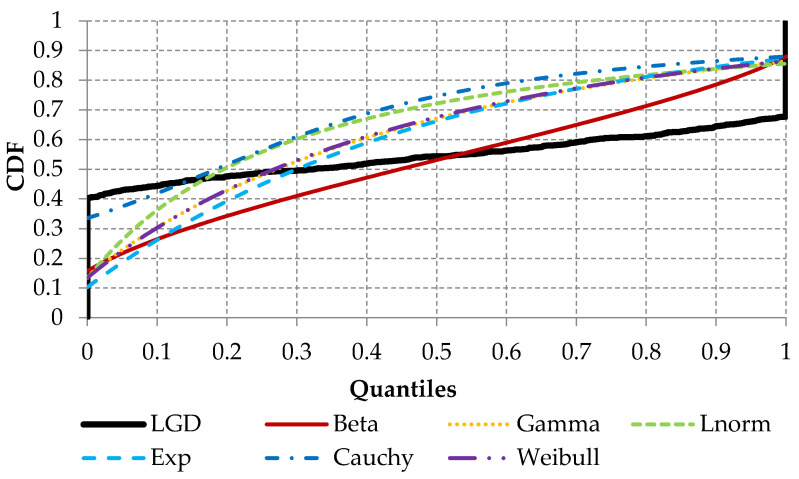
The cumulative distribution functions for the investigated parametric distributions.

In the next step, several parametric distributions were selected and tested using empirical *LGD* values. In the scope of the analysis, the following distributions were selected based on the following expert decisions: Beta, Gamma, Log-normal, Exponential, Cauchy and Weibull. The analyzed distributions were fitted using maximum likelihood estimation and goodness-of-fit statistics for each distribution; they were then computed and are presented below (Figure 4). By analyzing empirical (black solid line) and theoretical cumulative distribution functions, it can be stated that all considered parametric distributions do not reflect empirical *LGD* distributions very well, but, in terms of considered distribution, the Beta distribution (red solid line) seems to be “good enough”.

This was also confirmed by the goodness-of-fit statistics. For each statistic, the Beta distribution has the lowest values (See Table 2). Empirical *LGD* distribution is very difficult to reflect due to the fact that empirical distribution has two high peaks: one peak is connected with a zero value and second peak with a value of one.

In the analyzed data, we observed a high level of missing data. For 14 variables, the missing data ratio was higher than 30% (see Table 3). On the other hand, 142 variables were populated by 100% of the data. Missing values have an impact on the considered methods because they may result in a decrease in model performance and may lead to distorted results and incorrect final conclusions. In the literature, to overcome the missing data problem, several approaches are considered, as follows: (1) imputation using mean or median values; (2) imputation using the most frequent or zero values; (3) random values from a theoretical distribution or; (4) model imputation. From a theoretical perspective, the iterative imputation using Random Forests proposed by Stekhoven and Bühlmann [64] is especially interesting due to the following facts: (1) the proposed algorithm can handle any type of input data; (2) it requires as few as possible theoretical assumptions; (3) it uses non-parametric method that allows us to model complex interactions; (4) it avoids imputation with values not present in the original data; (5) it can manage high dimension problems.

Considering all mentioned cons, the iterative imputation method using Random Forests was applied to impute missing values in the analyzed data. Additionally, it was observed that for several variables the variance is very small or equal to zero. Due to the use of distance metrics in the next step, the variables with coefficient of variance equal to zero (10 variables) or less than 0.02 (only two variables) were excluded.

One goal of this paper is to compare the quantile regression model with the weighted quantile Regression Forests model. Weighted quantile Regression Forests are non-parametric models and can manage with high dimension problems and automatically select the best variables according to the specified criteria. The quantile regression model is classified as a parametric model and the structural model should be specified before estimation. To avoid the problem of variable selection using the considered methods and to concentrate our attention on model comparison, not business explanation, we decided to perform a clustering analysis to limit the number of variables in the next steps. The clustering of variables can be defined as a way to arrange variables into smaller clusters, i.e., groups of variables that are correlated to each other and that bringing the same information. The described approach can be used to reduce the time spent on dimension and variable selection [65]. Several methods were developed for the clustering of numerical variables. In this paper, we decided to perform a hierarchical clustering algorithm with a similarity index between variables, defined as a Spearman correlation coefficient. Finally, we decided to select only one variable from each of the 25 clusters. In summary, to reduce the number of features, we applied a variable selection procedure based on Algorithm 2.
**Algorithm 2.** Variable selection pseudocode.**input**: List of all explanatory variables X, distance function $dist\left(C_{i},C_{j}\right)$, correlation matrix (M), target variable (y)**output**: List of the best explanatory variables (B)1:     create empty cluster list C
2:     **for each**
j = 1 to p
**do**3:       create a cluster for each variable $C_{j}=\left\{X_{j}\right\}$
4:     **end**5:     calculate distance matrix D as 1−|M|
6:     **repeat**7:       $d_{i,j}^{min}$ = find smallest element $d_{i,j}$ in D for all $C_{i},C_{j}\in C$
8:       **if**
$d_{i,j}^{min}>0.9$
**then**9:         **break repeat**10:     **end**11:     merge cluster $C_{j}$ and cluster $C_{i}$
12:     update distance matrix D
13:   **end**14:   create empty MSE_list
15:   **for each**
j = 1 to p
**do**16:     MSE = estimate mean square error of j-th variable for prediction of y
17:     update MSE_list based on MSE
18:   **end**19:   **for each**
$C_{i}\in C$
**do**20:     select variable based on the MSE_list and add to B
21:   **end**22:   **return**
B


### 5.4. Model Comparison

This section presents the goodness-of-fit measures to compare the performance of each investigated model. The goodness-of-fit is performed considering the entire distribution and not only the mean value, since most research papers about *LGD* concentrate only on mean prediction. Mean prediction did not reflect the distribution of *LGD*. The estimates of the performance measures were produced with 10-fold cross-validation. All further results are presented as a 10-fold average with the standard errors of the estimates. Below, we enumerate and describe three measures and one plot which compare all the distributional characteristics of the *LGD*.

The first measure is the coefficient of determination $R^{1}$ (which is equivalent to the $R^{2}$ used in standard regression problems) separated for each quantile [3]:

(15) $$R^{1}\left(\alpha \right)=1-\frac{\sum_{i=1}^{n}\rho_{\alpha }\left(y_{i}-{\hat{Y}}_{i}\right)}{\sum_{i=1}^{n}\rho_{\alpha }\left(y_{i}-{\hat{Q}}_{\alpha }\left(y\right)\right)},$$

where ${\hat{Q}}_{\alpha }\left(y\right)$ is the α-quantile of realizations of the *LGD* and $\rho_{\alpha }$ is the asymmetric loss function defined as follows:

(16) $$\rho_{\alpha }\left(x\right)=\{\begin{array}{cc} \alpha x, & x\geq 0\\ \left(1-\alpha \right)\left|x\right|, & x<0 \end{array}.$$

where a higher value of $R^{1}$ represents a better fit for the specific quantile.

**Figure 5:**
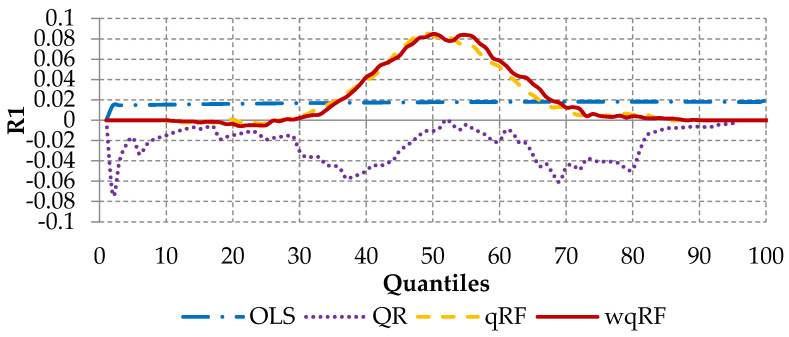
An average $R^{1}$ measure on test sample (10 folds).

Figure 5 shows the $R^{1}$ values over the entire distribution for each model for the test sample (10-fold average). Based on this figure it can be stated that the standard quantile Regression Forests (orange dashed line) and the weighted quantile Regression Forests (red solid line) dominate other methods. The superiority of these methods is especially given in the middle part of the distribution between the 30th and the 80th percentile. Quantile regression (dotted purple line) is “moderately well fitted” for percentiles between 15 and 25, 50 and 55, and above 85. Finally, what is most important for this article is the fact that it is proven that the wqRF model outperforms the standard qRF between the 55th and 72nd percentile.

The second measure used in this paper is the probability-probability (P-P) plot, which is a graphical picture representing the distributional accuracy. The P-P plot presents empirical and theoretical quantiles by fitting the following values against each other [3]:

(17) $$p_{1i}=\frac{i-0.5}{n}\;\mathrm{and}\;p_{2i}=\hat{F}\left(y_{i}\right).$$

where *LGD* values (i=1,…,n) are ordered to ensure monotonic increasing quantiles $\hat{F}\left(y_{i}\right)$.

**Figure 6:**
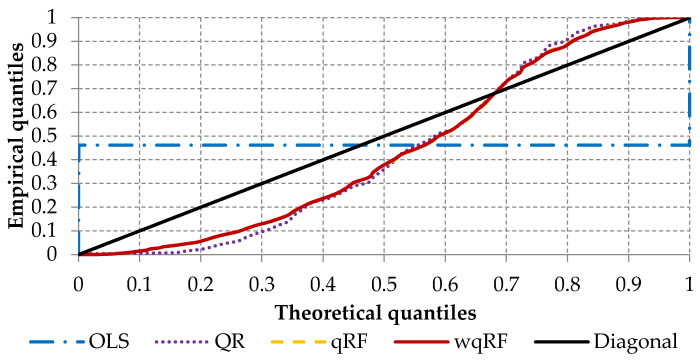
An average probability-probability (P-P) plot on test sample (10 folds).

The lower the difference between empirical and theoretical quantiles, the better the accuracy. Figure 6 shows the P-P plot. The reference is the bisector line, where the empirical quantiles match the theoretical quantiles.

Based on the P-P plot, it can be stated that the quantiles estimated by standard quantile Regression Forests (orange dashed line) are similar to the quantiles estimated by the weighted quantile Regression Forests (red solid line). Additionally, the quantiles estimated by those methods are “closer” to the theoretical quantiles than the quantiles estimated by quantile regression (dotted purple line). Moreover, to summarize the presented plot to one simple value, the following measures were calculated:

(18) $$\mathrm{HMI}=\frac{2}{n}\overset{n}{\underset{i=1}{\sum }}\left|p_{1i}-p_{2i}\right|\;\mathrm{and}\;\mathrm{HWMI}=\frac{2}{n}\overset{n}{\underset{i=1}{\sum }}{\left(p_{1i}-p_{2i}\right)}^{2}.$$

The Harmonic Mass Index (HMI) and the Harmonic Weighted Mass Index (HWMI) were determined by Hinloopen and van Marrewijk [66] as the mean absolute and mean squared difference compared to the perfect line [3]. The HMI and HWMI are standardized to values between zero and one and lower values of HMI or HWMI represent better accuracy. The values presented in Table 4 indicate that, for the HMI index, the best accuracy is achieved for the weighted quantile Regression Forests model, with a value equal to 0.151. In terms of the HWMI index, the accuracy for the weighted quantile Regression Forests is similar to the standard quantile Regression Forests (0.017).

The presented results confirmed that quantile regression can be used to model the *LGD* variable, with success. Similar results were presented by Krüger and Rösch [3], where the application of quantile Regression was compared to the other statistical methods. Additionally, our research showed that the quantile Regression Forest and the weighted quantile Regression Forests give better accuracy than quantile regression alone. It can be stated that non-parametric models perform better in comparison to parametric models. Similar results were presented by Qi and Zhao [4] and Bastos [21]. They considered only mean predictions, but their conclusions are similar to the conclusions presented here. A comparison of parametric and non-parametric models was also conducted by Hartmann-Wendels et al. [22]. They considered only the decision tree algorithm, but the results presented by the authors are similar to our results.

## 6. Conclusions

Loss given default is an important component in the Basel Accords. Using more advanced methods in comparison to those currently used gives the bank the possibility to calculate their regulatory capital in a more accurate way and may lead to a healthier and riskless allocation of capital. Findings from this research can help financial institutions when estimating *LGD* under the Internal Ratings Based Approach of the Basel Accords in order to estimate the downturn *LGD* needed to calculate the capital requirements. Most analytical parametric and non-parametric methods (such as ordinary least squares, decision trees, artificial neural networks, etc.) used for *LGD* modeling consider only the prediction of the mean, and do not consider the entire *LGD* distribution. This causes a serious problem since the empirical *LGD* distribution reflects the bimodal distribution, with peak values at the opposite sides, i.e., full loss (one value) or full recovery (zero value). To overcome this fact, this paper presented the weighted quantile Regression Forests model, built based on the data from one of the biggest banks in Poland. With our analysis, we confirm that predicting the future values of the loss given default is feasible and can be achieved with reasonable accuracy compared with the base models (first research question). This statement is supported by the results presented in Table 4 and Figure 5 and Figure 6. Our answer to the second research question is positive as well. The results proved that the weighted quantile Regression Forests are able to predict future *LGD* values with better accuracy than the benchmarking standard quantile Regression Forests. Finally, we have also empirically confirmed that both qwRF and qRF outperform other benchmarking methods including quantile regression and ordinary least squares models (third research question). The contributions of this article can be summarized as follows:

- Systematization of the knowledge regarding bimodal distribution, loss given default modeling and attempts to improve the Regression Forest algorithm;
- Application of the various modeling methods for loss given default problem;
- Incorporating of the weighting procedure in quantile Regression Forests.

Future work will be conducted in two ways, i.e., with a focus on business perspective and modeling methodology. In the first path, a similar analysis should be performed using other banks’ products. Our comparison was performed using only one product, the overdraft, so an interesting challenge would be to perform a similar analysis using another bank product. The incorporation of other macroeconomic variables should be thoroughly analyzed to provide insights about the analyzed models. In the second path, future work in this area should include extending this weighted ensemble framework to other modeling problems, including simple regression and multiclass classification. Moreover, we will try to apply a similar concept to other ensemble creation methods. Lastly, we intend to further investigate the performance and default settings of parameters in the context of the deviation and variance of the base models, with (potentially) both theoretical and empirical analyses.

## Figures and Tables

**Table 1:** Loss given default in the dataset and basic characteristics.

Characteristics	Min	P25	P50	Mean	P75	Max	Skewness	Kurtosis
**Value**	0.0000	0.0000	0.3413	0.4570	1.0000	1.0000	0.1699	−1.8048

**Table 2:** Goodness-of-fit statistics for the investigated parametric distributions.

Measure	Beta	Gamma	Log-Norm	Exponential	Cauchy	Weibull
Kolmogorov-Smirnov statistic	0.25	0.26	0.27	0.30	0.34	0.27
Cramer-von Mises statistic	16.50	19.54	20.84	23.82	28.05	20.15
Anderson-Darling statistic	110.57	128.27	135.54	165.02	170.07	132.79
Akaike’s Information Criterion	−504.92	493.61	539.62	521.19	2049.24	501.45
Bayesian Information Criterion	−494.82	503.71	549.72	526.24	2059.34	511.55

**Table 3:** Missing variables data ratio.

Group	0% Missing	0–2% Missing	2–5% Missing	5–10% Missing	10–16% Missing	16–20% Missing	20–30% Missing	30–90% Missing
**Number of missing variables**	142	57	27	5	16	17	14	14

**Table 4:** Goodness-of-fit statistics on test sample (10-fold).

Measure	OLS	QR	qRF	wqRF
**HMI**	0.645	0.169	0.153	0.151
**HWMI**	0.256	0.022	0.017	0.017
